# HEALTH LITERACY ACROSS PERSONALITY TRAITS AMONG OLDER ADULTS: CROSS-SECTIONAL EVIDENCE FROM SHARE

**DOI:** 10.1093/geroni/igac059.2376

**Published:** 2022-12-20

**Authors:** Valerie-Anne Ryser, Clément Meier, Sarah Vilpert, Jürgen Maurer

**Affiliations:** Swiss Centre of Expertise in the Social Sciences, Lausanne, Vaud, Switzerland; University of Lausanne, Lausanne, Vaud, Switzerland; University of Lausanne, Lausanne, Vaud, Switzerland; University of Lausanne, Lausanne, Vaud, Switzerland

## Abstract

**Introduction:**

Personality traits (PTs) - Neuroticism, Extraversion, Openness to experience, Agreeableness, Conscientiousness - are related to how older adults deal with health-related issues. However, little is known about the relationship between PTs and health literacy (HL). HL measures individuals’ ability to find, understand, appraise, and use health information to deal with health-related outcomes. Objectives: This research tries to understand better differences in HL across PTs in a nationally representative sample of adults aged 58 years and older in Switzerland. Method: Multivariable probit regressions to explore how respondents’ PTs are independently associated with HL after controlling individuals’ social, regional and health characteristics are based on a paper-and-pencil self-completion questionnaire (N= 1’555) administered as part of wave 8 (2019/2020) of SHARE in Switzerland. HL is measured using the short version of the European Health Literacy Survey questionnaire (HLS-EU-Q16), whose scores of dichotomized responses is grouped into two categories: inadequate and adequate HL. PTs are measured with the Big-Five inventory ten (BFI-10).

**Results:**

Results show that two out of five PTs are significantly associated with HL among older adults. Individuals who score higher on neuroticism and thus have a persistent tendency to experience negative emotions are more likely to have inadequate HL. More open individuals who are more prone to engage in self-examination are also more likely to have adequate HL.

**Conclusion:**

These findings call for targeted interventions, such as using adjusted health or eHealth information tools that would consider individuals’ PTs when designing health policies to improve HL in the population.

